# Exploring *N*^6^-methyladenosine (m^6^A) modification in tree species: opportunities and challenges

**DOI:** 10.1093/hr/uhad284

**Published:** 2023-12-29

**Authors:** Muthusamy Ramakrishnan, K Shanmugha Rajan, Sileesh Mullasseri, Zishan Ahmad, Mingbing Zhou, Anket Sharma, Subbiah Ramasamy, Qiang Wei

**Affiliations:** State Key Laboratory of Tree Genetics and Breeding, Co-Innovation Center for Sustainable Forestry in Southern China, Bamboo Research Institute, Key Laboratory of National Forestry and Grassland Administration on Subtropical Forest Biodiversity Conservation, School of Life Sciences, Nanjing Forestry University, Nanjing 210037, Jiangsu, China; Department of Chemical and Structural Biology, Weizmann Institute of Science, 7610001 Rehovot, Israel; Department of Zoology, St. Albert’s College (Autonomous), Kochi 682018, Kerala, India; State Key Laboratory of Tree Genetics and Breeding, Co-Innovation Center for Sustainable Forestry in Southern China, Bamboo Research Institute, Key Laboratory of National Forestry and Grassland Administration on Subtropical Forest Biodiversity Conservation, School of Life Sciences, Nanjing Forestry University, Nanjing 210037, Jiangsu, China; State Key Laboratory of Subtropical Silviculture, Bamboo Industry Institute, Zhejiang A&F University, Lin’an, Hangzhou 311300, Zhejiang, China; Zhejiang Provincial Collaborative Innovation Center for Bamboo Resources and High-Efficiency Utilization, Zhejiang A&F University, Lin’an, Hangzhou 311300, Zhejiang, China; State Key Laboratory of Subtropical Silviculture, Bamboo Industry Institute, Zhejiang A&F University, Lin’an, Hangzhou 311300, Zhejiang, China; Cardiac Metabolic Disease Laboratory, Department of Biochemistry, School of Biological Sciences, Madurai Kamaraj University, Madurai 625 021, Tamilnadu, India; State Key Laboratory of Tree Genetics and Breeding, Co-Innovation Center for Sustainable Forestry in Southern China, Bamboo Research Institute, Key Laboratory of National Forestry and Grassland Administration on Subtropical Forest Biodiversity Conservation, School of Life Sciences, Nanjing Forestry University, Nanjing 210037, Jiangsu, China

## Abstract

*N* ^6^-methyladenosine (m^6^A) in eukaryotes is the most common and widespread internal modification in mRNA. The modification regulates mRNA stability, translation efficiency, and splicing, thereby fine-tuning gene regulation. In plants, m^6^A is dynamic and critical for various growth stages, embryonic development, morphogenesis, flowering, stress response, crop yield, and biomass. Although recent high-throughput sequencing approaches have enabled the rapid identification of m^6^A modification sites, the site-specific mechanism of this modification remains unclear in trees. In this review, we discuss the functional significance of m^6^A in trees under different stress conditions and discuss recent advancements in the quantification of m^6^A. Quantitative and functional insights into the dynamic aspect of m^6^A modification could assist researchers in engineering tree crops for better productivity and resistance to various stress conditions.

## Introduction

RNA modifications that naturally occur in eukaryotic messenger RNA (mRNA), long non-coding RNA (lncRNA), ribosomal RNA (rRNA), transfer RNA (tRNA), small nuclear RNA (snRNA), small nucleolar RNA (snoRNA), circular RNA (circRNA), etc. have been shown to be an important biological mechanism for regulating RNA activity [1–4]. To date, more than 170 RNA modifications constitute the ‘epitranscriptome’ repertoire [5, 6]. Among the various RNA modifications, the functions of *N*^6^-methyladenosine (m^6^A), 5-methylcytosine (m^5^C), *N*^1^-methyladenosine (m^1^A), pseudouridine (Ψ), and 2′-*O*-methylation (Nm) are better documented than other modifications [2, 7]. m^6^A was first discovered in eukaryotic mRNAs and accounts for over 80% of all RNA methylation [8]. Compared with m^5^C, m^1^A, Ψ, and Nm, m^6^A is the most common modification in plants, and its function has been better elucidated in several plants, including *Arabidopsis*, maize, wheat, oats, and rice [9, 10].

The m^6^A modification varies among different plant tissues [2, 9, 11], and serves as a key switch for translation efficiency, nuclear retention, splicing, and RNA decay, through the recruitment of m^6^A-binding proteins [12, 13]. Moreover, the regulation of m^6^A is crucial for plant development under various stress conditions, including embryonic development, leaf initiation, shoot stem cell fate, trichome morphogenesis, flower transition, and root development [14–17]. Although m^6^A is evolutionarily conserved and plays important roles in cellular and biological processes [18, 19], the functional role of m^6^A modification in trees is less clear. Though the physiological functions of m^6^A in *Arabidopsis* have been well reviewed [2, 20, 21], reviews focusing on the function of m^6^A in trees are limited. In this review we summarize the biological functions of m^6^A in trees in response to biotic and abiotic stresses. In addition, we also summarize recent technologies capable of both detection and quantification of m^6^A.

## How m^6^A modifications are created and removed in trees

The m^6^A pathway involves three core RNA-binding proteins (RBPs), namely m^6^A writers (methyltransferases), m^6^A readers, and m^6^A erasers (demethylases), that collectively make m^6^A dynamic and reversible. These proteins are referred to as WERs (writers, erasers, and readers). Depending on downstream genes being targeted, m^6^A WERs can have different functions or the same function under different conditions [15, 22]. Although m^6^A WERs have been identified in both *Arabidopsis* and trees, the majority of these discoveries are restricted to *Arabidopsis*. This limitation restricts our comprehensive understanding of m^6^A modifications in trees.

**Figure 1:**
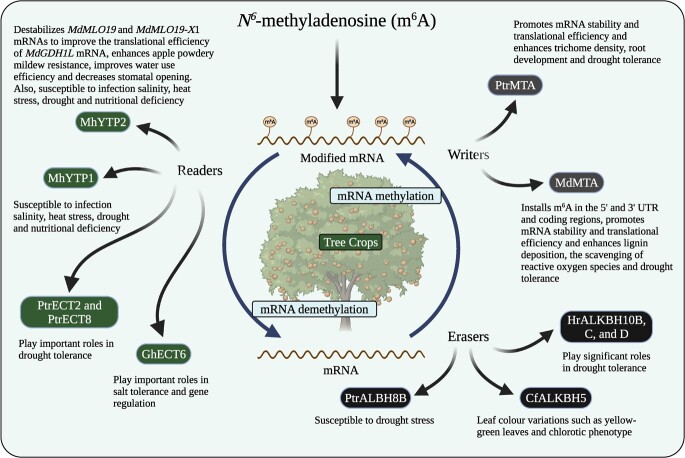
Schematic overview of the m^6^A writers, erasers, and readers so far identified and their known biological functions in trees. Created with BioRender.com.

Trees are characterized by their larger and more complex genomes and are perennial species exposed to different environmental conditions throughout their lifespan. Therefore, trees might exhibit different m^6^A modification patterns compared with *Arabidopsis*, thereby presenting a unique challenge in deciphering the intricacies of m^6^A modifications. However, the differences in modification pattern and its stoichiometry between trees and *Arabidopsis*, and the functions of WERs in trees remain unclear despite the identification of several WERs in tree species (Fig. 1). Furthermore, the economic and ecological importance of trees underscores the importance of elucidating m^6^A modifications in tree species. m^6^A modifications can significantly influence tree growth, development, and stress responses [19, 23, 24]. Consequently, understanding m^6^A in tree species is of paramount importance. Such an understanding will not only enrich our understanding of m^6^A modifications in trees but also provide insight into how this modification contributes to tree adaptation and resilience in different ecosystems. This perspective emphasizes the need to overcome the limitations of using *Arabidopsis* and highlights the potential implications for our understanding of m^6^A modifications in tree species. The following subsections focus on the functional significance of m^6^A WERs in tree crops.

### m^6^A writers

The m^6^A writer is a methyltransferase protein that adds a methyl group to adenosines. These m^6^A writer complexes were first identified in HeLa cells [25–27]. Later, orthologs of animal m^6^A writers, including MTA (an ortholog of METTL3, a SAM-binding protein) [28], MTB (an orthologue of METLL14, an mRNA target-binding protein) [29], and FIP37 (an orthologue of WTAP) [30], were identified in *Arabidopsis*. Besides this, *Arabidopsis* also contains the E3 ubiquitin-protein ligase Hakai (HAKAI), which is involved in the regulation of m^6^A writer components [29]. In rice, an alternative m^6^A pathway, mediated through ENHANCED DOWNY MILDEW 2 protein (EDM2), has been identified [31].

Recent studies reveal that mutants of m^6^A writer components, such as MTA, MTB, VIRILIZER (VIR), and HAKAI, show salt-sensitive phenotypes in an m^6^A-dependent manner. VIR-mediated m^6^A in mRNA was found to be correlated with mRNA stability by altering the length of 3′-UTR transcripts through alternative polyadenylation [32]. To date, only two studies have reported the function of m^6^A writers in trees. PtrMTA in poplar [33] and MdMTA in apple trees [24] have been identified as critical for m^6^A modification. The overexpression of *PtrMTA* increased drought tolerance by promoting trichome and root development in poplar [33]. MdMTA is important for drought tolerance [34], and this might prove to be helpful in the search for potential genes for developing stress-tolerant apple cultivars.

### m^6^A readers

The m^6^A reader proteins, which recognize m^6^A marks on target sites and regulate biological functions, were first discovered in animals [35, 36]. The YTH protein family functions as a group of specific reader proteins [37]. Depending on the type of YTH protein, m^6^A can affect mRNA metabolism in different ways, such as modulating stability, promoting translation, or affecting splicing [27].

In contrast to the extensive studies performed on YTH domain-containing proteins (YTHDs) in animals, only three evolutionarily conserved proteins of the C-terminal region (ECT) family have recently been functionally characterized as YTHD homologs in *Arabidopsis* [15, 16]. The m^6^A reader proteins mainly include ECT2, ECT3, ECT4, and CPSF30 [38]. YTH proteins are abundant in GL-3 (Royal Gala) apple, *Malus hupehensis* (Chinese crab apple) [39], cucumber [40], and rice [41]. In *Arabidopsis*, ECT2 controls proteasome activity, trichome morphology, and developmental timing and morphogenesis by fine-tuning mRNA stabilization. Moreover, ECT2, ECT3, and ECT4 relocalize to cytoplasmic foci during osmotic stress, whereas only ECT2 relocalizes to cytoplasmic stress granules during heat stress [16, 42, 43]. In addition, ECT1 and ECT2 also interact with the stress response protein *CALCINEURIN B-LIKE-INTERACTING PROTEIN KINASE1* (*CIPK1*) to facilitate the transmission of calcium signals into the nucleus during various external stimuli [44].

In apple, m^6^A reader *MhYTP2* overexpression enhances apple powdery mildew resistance by regulating the mRNA stability of *MdMLO19* and *MdMLO19-X1* and improving the translation efficiency of *glutamate dehydrogenase 1-like MdGDH1L* [45]. In addition, *MhYTP2* also improves water use efficiency by increasing the photosynthetic rate and water uptake by roots and decreasing stomatal opening through increased abscisic acid concentrations and activated ethylene signaling [46]. However, the overexpression of *MhYTP1* or *MhYTP2* in GL-3 (Royal Gala) apple led to higher sensitivity to infection caused by *Diplocarpon mali* (Marssonina apple blotch), salinity, and heat stress, and enhanced resistance to water-logging, chilling, drought, and nutritional deficiency [39]. Furthermore, the overexpression of *MhYTP1* and *MhYTP2* was found to promote leaf senescence in *Arabidopsis* and GL-3 (Royal Gala) apple, and fruit ripening in tomato [47]. In poplar 84K (*Populus alba* × *Populus glandulosa*), a fast-growing poplar hybrid, the expression pattern of m^6^A pathway genes was tissue-specific and there was differential expression in leaves, xylem, phloem, and roots [48].

### m^6^A erasers

m^6^A erasers, also known as demethylases, are responsible for removing the methyl groups from the methylated adenosine [49]. The first identified RNA demethylase, Fat mass and obesity-associated protein (FTO), catalyzes the demethylation of m^6^A in human mRNA [50]. Introducing human FTO to rice and potato increased crop yield and biomass by 50%. FTO stimulates root meristem, cell proliferation, and tiller bud formation and promotes photosynthetic efficiency and drought tolerance by mediating substantial m^6^A demethylation [51]. ALKBH (α-ketoglutarate-dependent dioxygenase homolog) proteins are another class of erasers that remove RNA methylation marks [52, 53]. Under normal and salt stress conditions, the m^6^A levels in *alkbh10b* mutants in *Arabidopsis* were higher than those of the wild type [54]. In addition to stress response, ALKBH proteins also perform various other functions. Depletion of m^6^A demethylase suppresses vegetative growth and floral transition by affecting the stability of the target mRNA [14]. ALKBH10B affects *Arabidopsis* floral transition and modifies the abscisic acid response during seed germination [14, 55]. ALKBH9B regulates m^6^A abundance on viral RNAs and is required for the vascular movement of Alfalfa mosaic virus in *Arabidopsis* [56]. Although many eraser proteins targeting specific methylation marks have been identified in model plants such as *Arabidopsis*, only three studies have reported m^6^A eraser proteins in trees under drought stress and for leaf color variations: HrALKBH10 in sea buckthorn [57], PtrALBH8B in poplar [58], and CfALKBH5 in *Catalpa fargesii* [59]. The level of activity of most ALKBH family members in trees has yet to be determined.

### Identification of RNA-binding sites for m^6^A writers, erasers, and readers

WERs associated with RNAs and their modifications play crucial roles in various cellular processes. Therefore, identification of binding sites for RBPs is important in order to understand how m^6^A modifications are generated and removed. To address this issue, TRIBE (Targets of RNA-binding proteins identified by editing) was introduced, which expresses RBP fused to the catalytic domain of the RNA-editing enzyme ADAR (ADARcd) *in vivo* to identify RBP binding sites [60]. ADARcd tags target RNA transcripts by converting A to I near the RBP binding sites. However, the editing efficiency is low, and the editing sequence is biased [61]. Thus, HyperTRIBE with the hyperactive mutation E488Q was incorporated into ADARcd to increase editing efficiency and reduce the sequence bias [62, 63]. Using HyperTRIBE, the targets of ECT2 and ECT3 were identified [64]. HyperADARcd in plants showed better performance than other RNA editing enzymes and identifies RBPs and their targets in a simple manner [65]. These methods can be used to identify m^6^A-associated targets of m^6^A writers, readers, and erasers in trees in future.

## Functions of m^6^A modification in trees under stress conditions and normal development

In a cellular context, different types of mRNA may have different amounts of m^6^A. For example, in *Arabidopsis* there are ~5000 mRNA transcripts that contain 0.5–0.7 m^6^A peaks per 1000 nucleotides or 0.7–1.0 m^6^A peaks per actively expressed transcript. m^6^A has a significant influence on nuclear processes such as splicing and epigenetic activity and it plays an important role in promoting cytoplasmic mRNA degradation, affecting the corresponding cellular processes and pathways [66]. Since mRNA metabolism regulates nuclear export, alternative splicing, translation, degradation etc., m^6^A has a profound impact on all mRNA-associated processes [67, 68]. Thus, m^6^A influences almost every stage of the mRNA life cycle [28, 69].

As in the case of mammals, m^6^A plays a crucial role in plant development and maintains circadian and seasonal rhythms in plants [14, 70, 71]. In the coding regions, m^6^A affects translational dynamics [72, 73], and in the 5′-UTRs it promotes cap-independent translation [74]. However, unlike in mammals, m^6^A in plants is enriched around the stop codon and within the 3′-UTRs [69]. Though m^6^A has been discovered in almost all plant species, more attention has been paid to the model plant *Arabidopsis* [71, 75] and crop plants such as maize [76], wheat [77], and rice [51, 78]. Only a few studies have investigated m^6^A in trees (Table 1), such as apple [79], poplar [33], tea [80], citrus [81], sea buckthorn [57], and moso bamboo [19, 82]. In the following subsections, we will discuss the functional significance of m^6^A in tree mRNA under stress conditions.

**Table 1:** Physiological functions of *N^6^*-methyladenosine (m^6^A) in trees.

**Tree species**	**Function and possible involvement in the stress response**	**Reference**
Apple (*Malus domestica*)	MdMTA, the m^6^A methyltransferase complex, is responsible for m^6^A development in mRNA under drought conditions. Transcribing mRNAs are involved in oxidative stress and lignin deposition. The m^6^A modification in mRNA promotes mRNA stability and translational efficiency of the genes in response to drought and oxidative stress.	[24]
Apple	Under drought stress, m^6^A modification changes gene expression in drought-responsive genes such as *HEAT SHOCK PROTEIN 60* (*HSP60*), *JASMONATE-ZIM-DOMAIN PROTEIN 3* (*JAZ3*), *Scarecrow-Like 1* (*SCL1*), and *ETHYLENE RESPONSE FACTOR1* (*ERF1*).	[79]
Apple	Overexpression of the m^6^A reader *MhYTP2* increases mRNA m^6^A and regulates the mRNA stability of *MdMLO19* and the translational efficiency of antioxidant genes, resulting in resistance to powdery mildew in apples.	[45]
Chinese crab apple (*Malus hupehensis*)	*MhYTP1 and MhYTP2* were induced by methyl jasmonate and salicylic acid, and their overexpression made apple trees more susceptible to Marssonina apple blotch, heat stress, and high salinity, and more resistant to oxygen and nutrient deficiency. The promoter regions contain many stress-related *cis*-elements.	[39]
Poplar (*Populus trichocarpa*)	Overexpressed *PtrMTA* (methyltransferase) participates in m^6^A formation, improves drought tolerance, increases trichome density, and results in a better root system.	[33]
Poplar	m^6^A sites were mainly enriched in the coding regions and the 3′-UTRs and associated with drought-induced genes. PtrMTA transcripts showed a positive correlation with the protein content. Cellulose- and lignin-related genes in response to drought stress were associated with m^6^A ratio, and m^6^A interacted with poly(A) tail length (PAL) and polyadenylation.	[58]
Cotton (*Gossypium hirsutum*)	m^6^A is dynamic under salt stress. Genes containing m^6^A are differentially expressed in response to salt stress. m^6^A reader protein regulates salt tolerance.	[83]
Sea buckthorn (*Hippophae rhamnoides*)	The m^6^A modification genes are associated with metabolic biosynthesis. Three m^6^A demethylases, *HrALKBH10B*, *HrALKBH10C*, and *HrALKBH10D*, were significantly upregulated under drought stress. m^6^A demethylase genes decrease m^6^A methylation during drought stress.	[57]
Tea (*Camellia sinensis*)	m^6^A regulatory genes are driven by whole-genome duplication and segmental duplication events. m^6^A duplicated regulatory gene pairs evolved by purifying selection. Sequence variation of the regulatory genes contributes to m^6^A functional diversification. Regulatory genes are differentially expressed under stress conditions and in tea-withering stages. RNA methylation and DNA methylation develop negative feedback through interaction with other regulatory genes.	[80]
Citrus (*Citrus grandis*)	Comprehensive analysis of m^6^A regulatory genes reveals different expression patterns during different growth stages. The genes are divided into writers, erasers, and readers and are distributed across nine chromosomes. The domain structures are diverse among the m^6^A enzymes.	[81]
Moso bamboo (*Phyllostachys edulis*)	m^6^A sites enriched at different growth stages during rapid growth were higher in 2-m shoots than in 4-m shoots of the 18th internode, enriched mainly in the 3′-UTRs, and maintained the stability of the transcripts associated with rapid growth.	[19]
Moso bamboo	A novel method was developed to detect m^6^A, and m^6^A sites may also drive the translation of circularized transcripts expressed under GA_3_ treatments.	[82]
Moso bamboo	Decreased m^6^A level in the 3′-UTR region and increased m^6^A level in the coding region (as observed in genes such as *PedMKK3* and *PedMTA*) promoted the lateral root growth and development, increased expression of m^6^A writers, and increased expression of exon junction complexes such as *MAGO*, *Y14*, and *EIF4A-III*.	[23]
Maiyuanjinqiu derived from *Catalpa fargesii*	m^6^A in the 3′-UTRs showed a negative correlation with mRNA abundance, m^6^A pathways were related to photosynthesis and stress response, and increased m^6^A in yellow-green leaves decreased *CfALKBH5* gene expression and was associated with a chlorotic phenotype.	[59]

### m^6^A response to biotic stress in trees

In apples*,* the overexpression of *MhYTP2* increased m^6^A in mRNA and enhanced the translational efficiency of antioxidant genes, in the process improving resistance to powdery mildew [45]. *MhYTP2*-induced m^6^A modifications in the exon regions destabilized associated mRNAs, whereas m^6^A modifications in untranslated regions were positively correlated with mRNA abundance and improved powdery mildew resistance in apple trees through the rapid degradation of bound mRNAs of *MdMLO19* and *MdMLO19-X1* and the translation efficiency of the glutamate dehydrogenase-1-like gene, *MdGDH1L* [45]. In another study, in GL-3 (Royal Gala) apples, the overexpression of *MhYTP1 and MhYTP2* (m^6^A readers) resulted in increased susceptibility to Marssonina apple blotch. Moreover, several *cis*-acting elements related to biotic and abiotic stresses were identified in the promoter regions of *MhYTP1* and *MhYTP2*. Therefore, these two genes showed inducibility in response to various stress treatments and are actively involved in various stress responses [39]. Furthermore, it will be useful to investigate whether m^6^A has longer-term effects in apple species than in annual plants. While m^6^A in cereal crops may be temporary and present only during specific seasons, m^6^A in tree mRNA could potentially promote long-term stress responses. However, further evidence is required to support this hypothesis.

### m^6^A response to drought stress in trees

In response to drought stress in apple trees, the presence of m^6^A has been shown to affect both mRNA stability and the translational efficiency of drought- and oxidative stress-related genes [24]. In addition, *MdMTA* is involved in oxidative stress and lignin deposition under drought conditions [24]. A recent study conducted on apple seedlings revealed that m^6^A modulates drought response genes, including *HEAT SHOCK PROTEIN 60* (*HSP60*), *JASMONATE-ZIM-DOMAIN PROTEIN 3* (*JAZ3*), *Scarecrow-Like 1* (*SCL1*), and *ETHYLENE RESPONSE FACTOR1* (*ERF1*), which are related to drought tolerance [79]. In sea buckthorn, drought stress significantly induces the expression of three m^6^A demethylases (*HrALKBH10B*, *HrALKBH10C*, and *HrALKBH10D*), suggesting that decreased m^6^A methylation plays important roles in drought tolerance in sea buckthorn [57]. In poplar, Poplar methyltransferase (*PtrMTA*), m^6^A erasers (*ALBH8B*), and m^6^A readers (*ECT2* and *ECT8*) were upregulated under drought stress. The m^6^A ratio was associated with cellulose- and lignin-related genes in response to drought stress and reduced transcription and translation levels, suggesting that m^6^A represses wood formation under drought stress [58]. In another study, the overexpression of *PtrMTA* increased the root system, lignin deposition, and the scavenging of reactive oxygen species, leading to enhanced drought tolerance in poplar [33].

### m^6^A response to salt stress in trees

In cotton, m^6^A deposition was positively correlated with gene expression under salt stress [83]. m^6^A prevents RNA cleavage in stress-responsive transcripts and stabilizes mRNAs under salt and osmotic stresses [84]. Furthermore, m^6^A and RNA-associated secondary structure in *Arabidopsis* increased mRNA stability and ultimately protein levels during salt stress [85]. Rice stress tolerance requires the m^6^A-YTH system [86]. *GhECT6*, a YTH domain gene in cotton, plays an important role in salt tolerance [83]. In citrus, the *cis*-acting elements of 26 m^6^A regulatory genes encoding the regulatory proteins are related to the abscisic acid (ABA)-responsive element (ABRE) [81]. In tea, downregulation was observed in all m^6^A writer genes under drought treatment [80].

### m^6^A response to leaf color variations

Analysis of m^6^A composition in mRNA derived from leaves with different colors revealed varying correlation with gene expression in Maiyuanjinqiu and *C. fargesii* [59]. Maiyuanjinqiu is a new natural variety with yellow-green leaves derived from *C. fargesii*, the Chinese bean tree with green leaves. Increased m^6^A and decreased *CfALKBH5* gene expression (an m^6^A eraser) were positively correlated with yellow-green leaves, suggesting a chlorotic phenotype. In contrast, m^6^A levels were significantly lower in the seedlings of *C. fargesii* (green leaves) than in those of Maiyuanjinqiu (yellow-green leaves), further supporting a positive correlation between increased m^6^A, decreased *CfALKBH5* gene expression, and the occurrence of a chlorotic phenotype. This implies that m^6^A levels may serve as a crucial epitranscriptomic marker for identifying leaf color variations in trees. Furthermore, m^6^A enrichment in the 3′-UTR was negatively correlated with global gene expression, and m^6^A-containing mRNAs were related to photosynthesis, pigment biosynthesis and metabolism, oxidation–reduction, and stress response [59]. This underscores the potential impact of m^6^A on broader physiological pathways related to leaf color variations in trees.

### m^6^A response in growth and development

In poplar, the overexpression of *PtrMTA*, which colocalized with *PtrFIP37* in the nucleus, led to increased trichome density and a more developed root system compared with the wild type. Furthermore, the m^6^A levels in the roots were higher than those of the wild type, indicating that m^6^A formation affects the development of trichomes and the root system, thereby enhancing drought tolerance [33]. In moso bamboo, which grows rapidly (up to 114.5 cm per day), m^6^A regulates the rapid cell division and elongation of each internode [87]. The m^6^A levels were higher in 2-m shoots than in 4-m shoots of the 18th internode, suggesting that m^6^A was slowly demethylated during rapid growth. m^6^A was also found to maintain the stability of mRNAs of the genes related to lignin biosynthesis during rapid growth [19]. Additionally, m^6^A modifications drive the regulation of circularized transcripts in moso bamboo seedlings treated with gibberellic acid (GA_3_) [82]. A recent study by the same group revealed the importance of m^6^A in moso bamboo root development [23]. This study observed that reducing m^6^A levels in the 3′-UTR regions while increasing them in the coding region, as seen in genes like *PedMKK3* and *PedMTA* due to the RNA methylation inhibitor (DZnepA), led to increased lateral root growth. This coincided with elevated gene expression, an increased full-length ratio, enhanced proximal poly(A) site utilization, and a shorter poly(A) tail length. Notably, common motifs in this process were AAACA and AAACT [23]. These findings underscore the indispensable role of m^6^A in moso bamboo development. A summary of all recent studies describing the functional role of WREs in tree crops is listed in Table 1.

## Emerging technologies to identify and quantify nucleotide-specific m^6^A

Many technological advances have helped to detect RNA modifications and uncover new functions and regulators involved in RNA modifications [88]. In the early stages, chromatographic methods, including high-performance liquid chromatography–mass spectrometry (HPLC–MS), thin-layer chromatography (TLC), and gas chromatography (GC), have been used to identify methylated nucleotides. HPLC–MS, dot-blot, and high-performance liquid chromatography coupled to triple-quadrupole mass spectrometry (LC–MS/MS) [89–91] are reliable and provide quantitative information on RNA modifications, making them suitable for analyzing a wide range of biological samples [92]. However, precise identification of RNA modification sites is essential for understanding the interaction between RNA functions and regulatory mechanisms.

**Figure 2:**
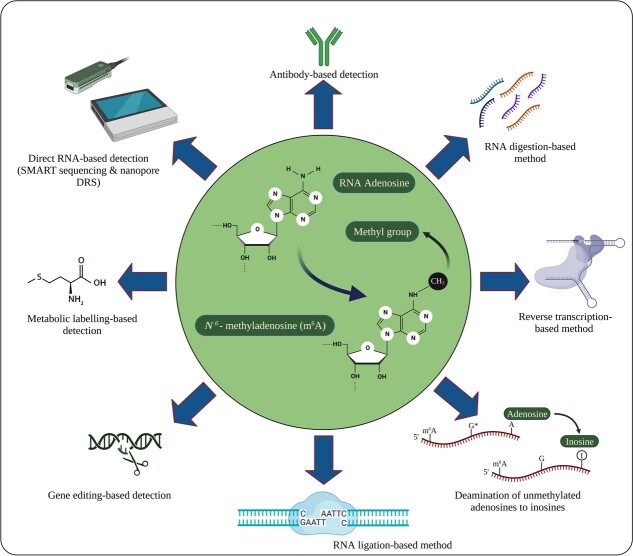
Different methods for m^6^A detection and profiling. Created with BioRender.com.

To date, there are numerous methods for profiling m^6^A modifications, including antibody-based immunoprecipitation, digestion-based detection, m^6^A sensing-reverse transcription-based detection, ligation-based detection, gene editing-based detection, metabolic labeling, and direct RNA-based detection (Fig. 2). The advantages and disadvantages of each method for m^6^A detection are listed in Table 2. Due to the word limit, only recent methods such as digestion-based detection, deamination of unmethylated adenosines, cryo-electron microscopy (cryo-EM), and nanopore direct RNA sequencing (DRS) are briefly described in the following sections, and the other methods are briefly described in Supplementary Data Appendix S1.

### Digestion-based detection

Nucleotides with or without m^6^A modification have different chemical and physical properties. Therefore, a liquid chromatography–mass spectrometry (LC–MS)-based approach has become the standard method for the quantification of RNA modifications by single nucleotide digestion and ultraviolet detection [93, 94]. RNase T1 and RNase A are commonly used to enzymatically degrade nucleic acids into single nucleotides. LC–MS compares single nucleotides with standard nucleotides to quantify m^6^A. The method features excellent selectivity, sensitivity, and simplicity. However, this method cannot determine the position of m^6^A in RNA molecules. Furthermore, enzymatic digestion of RNA into single nucleotides loses the sequence context [95, 96].

To address this issue, a strategy of targeted RNA fragmentation using specific enzymes along with LC–MS/MS analysis, similar to the methods utilized in proteomics, was implemented [97, 98]. However, the low abundance of modified mRNA made single-nucleotide resolution detection challenging. Another method for the accurate and quantitative identification of mRNA is site-specific cleavage and radiolabeling, followed by ligation-assisted extraction and TLC (SCARLET). SCARLET has the capability to detect low levels of m^6^A modification at specific sites and is more sensitive compared with methylated RNA immunoprecipitation sequencing (MeRIP-Seq)–qPCR. However, SCARLET also requires expensive chemicals and time-consuming processes, including many enzyme transformations and separations [99].

**Table 2:** Working principle, advantages, and disadvantages of methods for the identification, quantification and localization of m^6^A.

**Detection methods for m** ^**6**^ **A profiling**	**Working principle**	**Advantages**	**Disadvantages**
Antibody-based detection	Use of antibody enrichment followed by RNA sequencing	Identification of m^6^A from total RNA or mRNA	Library preparation, poor reproducibility, and no site-specific identification
Photo-crosslinking-assisted sequencing (PA-m^6^A-seq)	Incorporating photoactivatable nucleosides and then crosslinking mRNA at protein binding sites using light followed by m^6^A sequencing	Accurate identification of m^6^A sites and the best method for cultured cells	Requirement for cell pre-treatment, inconsistent quantification, and errors in transition sites and in exact positions
Reverse transcription-based method	Combination of reverse transcriptase and polymerases followed by RNA sequencing	Lower error rate in m^6^A identification, accurate site recognition, and no use of antibodies	Unquantifiable blocking effect, false-positive results, unable to detect in 5′-UTRs, and database limitations
Digestion-based detection	Digests into single nucleotides with RNase T1 and RNase A, followed by LC–MS, LC–MS /MS, TLC, SCARLET or MAZTER-seq and m6A- REF-seq	Precise quantification of m^6^A from nucleotides, nucleosides, and bases at low levels, identification at ACA sites and selectivity, sensitivity, and simplicity	Unable to detect exact positions and low abundance of m^6^A, loss of sequence context, requirement for expensive chemicals and many enzymes, and time-consuming processes
Ligation-based detection	Ligation of RNA templates using DNA probes ligated with T3 DNA ligase and amplification of ligated nucleotides	Accurate detection and quantification at one nucleotide resolution and higher specificity even in low abundance RNA in real biological samples	No genome-wide identification and accurate quantification, false-positive results, time-consuming processes, and requirement for PCR amplification
Gene editing-based detection	Conversion of C to U at targeted RNA sites using a base editing approach by fusing APOBEC1 (cytidine deaminase enzyme for RNA editing) to the m^6^A-binding YTH domain	Identification of global m^6^A in cells and single cells at low levels of RNA, isoform-specific m^6^A patterns, and m^6^A RNA-binding proteins, and antibody-free technique	False-positive results, requiring multiple molecular techniques (e.g. cloning and transfection), expensive chemicals and many enzymes, and time-consuming processes
Metabolic labeling-based detection (m^6^A-label-seq)	Feeding cells with m^6^A methyltransferase to generate and transfer methyl groups to specific mRNA adenosine to convert m^6^A to a^6^A by native m^6^A methylation enzymes	Detection of m^6^A at single-base resolution, detection of clustered m^6^A sites, and localization of nascent m^6^A in the nucleus	Requirement for several molecular techniques, expensive chemicals and many enzymes, and time-consuming processes
Direct RNA-based detection	Direct RNA sequencing (native/full-length RNA molecules in their native context) with Oxford Nanopore sequencing	Direct detection and quantification of m^6^A on native/full-length RNA molecules at single-base resolution, detection of novel m^6^A sites and no requirement for reverse transcription, amplification, antibodies, digestion, ligation, library preparation etc.	High error rates, database limitations in data analysis, unable to distinguish m^6^A from other modifications, and the requirement for computationally intensive tools
Cryo-EM	Imaging modified nucleotides within cellular conditions using cryo-EM with high resolution and 3D representation	Direct visualization of m^6^A nucleotides in macromolecular structures such as the ribosome, accurate structure-based rRNA and deep mapping	Ribosomes are prepared from a large quantity of cells, collecting cryo-EM data requires very expensive instruments and microscopes, and solving the structure takes a longer time
Deamination of unmethylated adenosines (similar to bisulfite sequencing)	Deamination of unmethylated adenosines to inosines followed by RNA sequencing	Transcriptome-wide absolute quantification of m^6^A at single-base high resolution, unbiased, and convenient	Requirement for multistep library preparation, short-read sequencing, inability to detect m^6^A interactions with other modifications, and need for improvement for standard applications of m^6^A

The ACA sequence-specific RNA endoribonuclease MazF, which was discovered in 2017, is sensitive to m^6^A and cleaves RNA only at the ACA sequence motif [100]. This characteristic allows the detection of m^6^A demethylase and methyltransferase activities. Normally, a reduction in MazF cleavage efficiency can indicate the presence of m^6^A residues within the ACA motif. To map m^6^A at single-nucleotide resolution with systematic quantitative profiling, MAZTER-seq (RNA digestion via m^6^A sensitive RNase) and m^6^A-REF-seq (m^6^A-sensitive RNA-endoribonuclease-facilitated sequencing) have also been developed using MazF [101, 102]. However, this method does not identify other known motifs as the enzyme only targets ACA sites. Thus, the universality of this method is limited, making it challenging to quantify m^6^A in all DRACH motifs, despite the availability of appropriate bioinformatics tools and resources for MAZTER-seq and m^6^A-REF-seq.

### Direct RNA-based detection

The direct RNA-based detection method provides significant advantage for quantitative m^6^A modification as it does not require reverse transcription, amplification, antibodies, digestion, ligation, etc. [103]. Nanopore DRS, a promising technology, can detect various RNA modifications in full-length RNA molecules [104]. During DRS, modified nucleotides and unmodified nucleotides emit different signal intensities when RNA molecules pass through the nanopore. This allows m^6^A identification using either comparative or supervised approaches. Nanopore m^6^A detection can be classified into two categories: the electrical signal and the basecalling error. Over the past 3 years, several algorithms and software packages (Tombo, EpiNano, DiffErr, ELIGOS, DRUMMER, MINES, XPore, Nanom6A, nanoDoc, Yanocomp, etc.) have been developed for the analysis of direct RNA nanopore sequencing [105–107]. Tombo, ELIGOS, DRUMMER, Yanocomp, xPore, nanoDoc, and Nanocompore use a comparative approach to distinguish m^6^A modification by comparing with control samples without m^6^A modifications [such as knockout or knock-down of m^6^A writer enzyme or *in vitro* transcribed (IVT) RNAs] [108]. On the other hand, MINES and Nanom6A use a supervised approach that utilizes training data or experimental protocols to identify m^6^A modification and overcome the limitations of the comparative approach. However, the supervised approach is limited to a specific nucleotide content. The advantages and disadvantages and the basic classification and GitHub repository of these packages are available in Supplementary Data Table S1.

**Figure 3:**
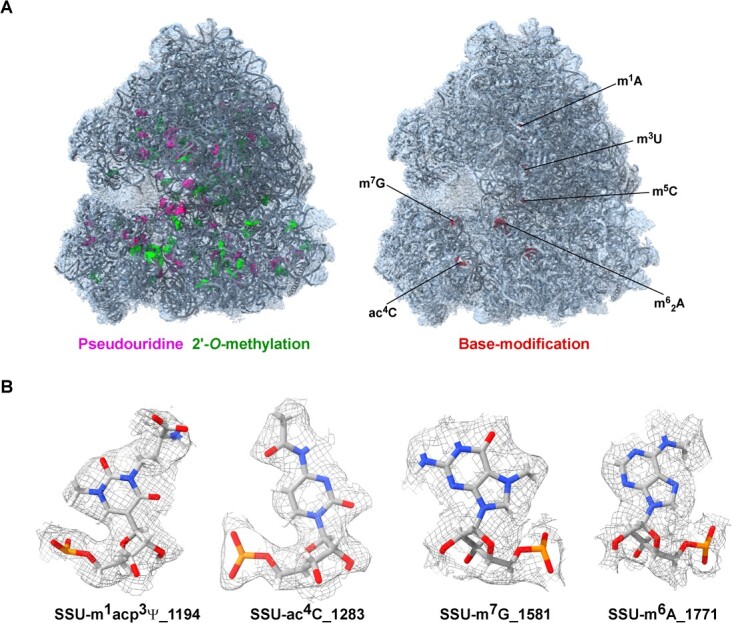
Detection and visualization of rRNA modification in plants. **A** Distribution of pseudouridine (Ψ) and 2′-*O*-methylation (Nm) in the tomato ribosome. The cryo-EM model and map are presented for the entire ribosome. **B** Cryo-EM density map of selected rRNA modification in plant ribosomes. The cryo-EM data ware derived from Cottilli *et al*. [114], deposited in PDB (PDB ID-7QIZ and EMD-14004).

EpiNano predicts m^6^A from DRS datasets by increasing mismatches and decreasing basecalling. However, it does not distinguish between m^6^A and other modifications such as m^1^A [109, 110]. MINES is another tool that provides the qualitative profiles of m^6^A sites, but it can only recognize m^6^A sites in four specific contexts: GGACA, GGACT, GGACC, and AGACT [111]. On the other hand, the Nanom6A pipeline utilizes an Extreme Gradient Boosting (XGBoost) model based on ion current signals from DRS to identify and quantify a transcriptome-wide m^6^A at single-base resolution with high accuracy [112]. This method also presents the m^6^A quantification of an individual transcript in different samples. In particular, in poplar transcripts [112] this method identified thousands of m^6^A sites enriched near the stop codon and 3′-UTR regions, as well as alternative polyadenylation sites. This suggests that Nanom6A is capable of conducting a transcriptome-wide analysis of m^6^A modifications and helps to understand the role of m^6^A in stabilizing highly expressed transcripts in genes associated with wood formation in tree species [112]. DiffErr (differr_nanopore_DRS) detects transcriptome-wide m^6^A using basecalling error [71]. Nanocompore, a robust and flexible analysis system with several unique features, was introduced to identify m^6^A with positional accuracy without the need for a training set. This method modifies the current signal and uses a model-free approach to compare m^6^A with different samples at single-molecule high resolution [106]. Recently, m6Anet, a neural-network-based method, has been reported for the quantification of m^6^A from DRS. However, m^6^A detection is still challenging due to the high error rates of DRS, which complicates the analysis of nanopore sequencing data. Comparison of 10 different tools used for identification of m^6^A from DRS data reveals that integrating analyses from multiple tools significantly enhances the effectiveness [113]. Further studies will aim to improve the ability for m^6^A detection based on nanopore DRS and algorithms in future.

**Figure 4:**
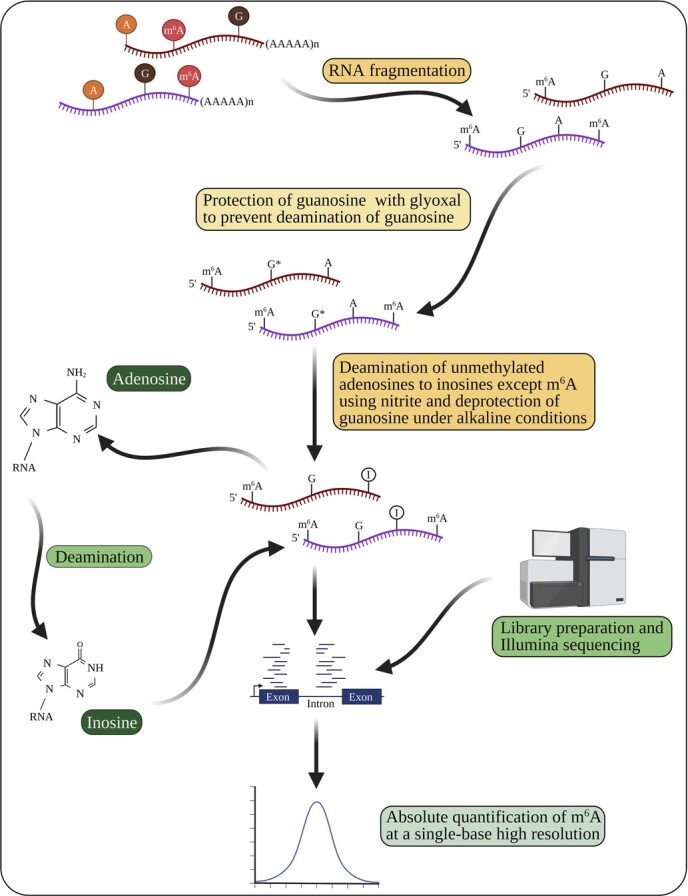
Deamination of unmethylated adenosines to inosines for a transcriptome-wide absolute quantification of m^6^A using GLORI (glyoxal- and nitrite-mediated deamination of unmethylated adenosines). This method consists of three steps: guanosine protection, adenosine deamination, and guanosine deprotection, followed by sequencing and absolute quantification of m^6^A. The schematic representation is based on Liu *et al*. [117] and Jones *et al*. [118]. Created with BioRender.com.

### Cryo-electron microscopy

Recent advancements in single-particle cryo-EM have enabled direct visualization of modified nucleotides in macromolecular structures like the ribosome [114]. A recent study using a 2.38-Å resolution cryo-EM reconstruction of the tomato ribosome was able to assign density for rRNA modifications [114]. The analysis of these ribosomes revealed 89 rRNA modifications, including Ψ, Nm (Fig. 3A), and base modifications such as m1acp3Ψ, *N*^4^-acetylcytidine (ac^4^C), *N*^7^-methylguanosine (m^7^G), and m^6^A (Fig. 3B). Although the method is not considered quantitative, this method provides nucleotide-specific detection and also could be used to study the function of RNA modification in the local and global structure of the RNAs they localize.

### Deamination of unmethylated adenosines

Bisulfite sequencing is widely regarded as the most reliable method for measuring DNA methylation at individual nucleotide resolution [115]. NOseq (amplicon sequencing evaluation method for RNA m^6^A sites after chemical deamination), similar to bisulfite sequencing, was the first method developed to detect m^6^A in RNA by chemical deamination (A to I). However, this method also introduces the G-to-X effect (deamination of guanosine into xanthosine) [116], which complicates transcriptome-wide studies. Recently, GLORI (glyoxal and nitrite-mediated deamination of unmethylated adenosines) has been developed for the absolute quantification of m^6^A at a single-base high resolution. GLORI consists of three steps: preventing deamination by protecting guanosine with glyoxal, deaminating all unmethylated adenosines except m^6^A to inosines using nitrite, and deprotecting guanosine under alkaline conditions (Fig. 4). The reverse transcription of inosines to guanosines allows comprehensive detection and stoichiometric quantification of m^6^A in mRNA fragments. GLORI has detected >175 000 m^6^A sites in HEK293T cells at a single-base resolution [117, 118]. Another method, evolved transfer RNA adenosine deaminase (TadA)-assisted m^6^A sequencing (eTAM-seq), is an enzyme-assisted sequencing method, which detects and quantifies site-specific m^6^A by inducing deamination of global adenosines to inosines using a hyperactive TadA variant. eTAM-seq facilitates transcriptome-wide m^6^A profiling without deep-sequencing by preserving RNA integrity from limited input samples [119]. Both GLORI and eTAM-seq are unbiased and convenient methods for the absolute quantification of m^6^A, with the potential to become a gold standard for m^6^A profiling.

### Databases for RNA modifications

Numerous RNA databases have also been developed to deposit RNA modifications. These RNA databases facilitate various tasks, such as RNA methylation site detection, motif discovery, differential RNA methylation analysis, and functional analysis. Currently, the MODOMICS database comprises a comprehensive list of RNA modifications discovered in all types of RNA molecules across all life kingdoms [120, 121]. Several other databases, such as RNAmod, also exist to catalog the RNA modification in model plants such as *Arabidopsis* (Supplementary Data Table S2). RNAmod is an interactive, web-based database designed for the functional annotation of mRNA modifications [122]. The RNAMDB database serves as a focal point for 109 known naturally occurring RNA modifications and continues to expand each year with new additions [123]. The m6Avar database contains 1 678 126 variants associated with nine types of RNA modifications, including m^6^A [124]. RNAWRE [125] and M6A2Target [126] are two dedicated databases for WERs, while RMBase is a comprehensive database for RBPs [127]. REPIC [128] and m6A-Atlas [129] are two dedicated databases for m^6^A and its site-specific interactions. DirectRMDB is the first database based on Oxford Nanopore Technologies, covering 16 types of modifications across 25 species [130]. However, there is currently no database that catalogs RNA modifications in tree species. Therefore, it is crucial to develop databases that provide information on mRNA modifications in tree species.

## Differences and similarities between *Arabidopsis* and trees with respect to m^6^A

Due to the contrast between the life cycles of *Arabidopsis*, a short-lived and small model plant, and tree species, long-lived and larger plants, differences in m^6^A patterns and stoichiometry are likely to exist. However, the precise functions of m^6^A in trees remain unexplored. This raises a crucial question: how does m^6^A modification differ between *Arabidopsis* and tree species in terms of functions, frequency of occurrence, and other relevant aspects? Thus, a comparative analysis of m^6^A modification between tree species and *Arabidopsis* would provide valuable insights into their similarities and differences. Therefore, through the analysis of m^6^A-related genes, their promoters, and their amino acids in trees and *Arabidopsis*, this review observed the differences and similarities in m^6^A WERs between these two species. A concise overview of the gene structure of m^6^A WERs in trees and *Arabidopsis* is listed in Supplementary Data Table S3.

### Specificity of m^6^A in tree species

*Distinct repertoire of m6A WERs.* Tree species possess a unique repertoire of m^6^A WERs compared with *Arabidopsis*, playing crucial roles in regulating m^6^A levels and patterns. For instance, in poplar the number of genes involved in the m^6^A pathway is significantly higher, comprising 61 m^6^A genes, including 14 m^6^A erasers, 14 m^6^A writers, and 33 m^6^A readers, in contrast to *Arabidopsis*, which has 28 m^6^A genes [48].

*Diverse functional roles.* m^6^A modifications in tree species are involved in a broad spectrum of biological processes (Fig. 1). While some functions may overlap with *Arabidopsis*, tree species exhibit distinct m^6^A-mediated regulatory mechanisms tailored to their unique life cycles and stress conditions [2, 45]. For instance, in *Arabidopsis* the m^6^A writer VIR and the m^6^A eraser ALKBH10B regulate salt tolerance, promoting seedling growth and seed germination [32, 54, 55]. In contrast, the m^6^A writer MTA plays a role in drought tolerance in apple and poplar [24, 33], while the m^6^A reader YTP2 regulates powdery mildew resistance in apple [45].

*Distinct target genes.* Although the primary function of WERs is to add methyl groups to adenosine residues, the specific target genes and biological consequences of m^6^A could differ between *Arabidopsis* and trees [2]. For instance, in apple, MhYTP2 targets *MdMLO19* (known to influence apple powdery mildew susceptibility) and antioxidant genes, reflecting the specific roles in tree species [45].

*Higher ratio of m6A.* Due to the higher abundance and activity of WERs in tree species [2, 48], the m^6^A ratio appears to be higher in tree species compared with *Arabidopsis*, suggesting that the m^6^A ratio may be linked to changes in the mRNA stability and translation efficiency of specific genes and may exert long-term effects in trees, aligning with their perennial life cycle and distinct developmental stages.

*Dynamic regulation of m6A.* Despite their evolutionary conservation across plant taxa, global and individual m^6^A levels display dynamic regulation in response to various stresses, exhibiting distinct patterns between tree species and *Arabidopsis* [2]. For instance, global m^6^A levels increase under salt stress in *Arabidopsis* [32], while they decrease under drought stress in sea buckthorn [57]. Interestingly, global m^6^A levels remain unchanged in apple under drought stress [24, 79], underscoring the dynamic and stress- or species-specific nature of m^6^A modifications. This dynamic regulation is likely mediated by altered expression of m^6^A WERs.

*Transcript-specific m6A.* While m^6^A modifications can occur on various transcripts, the extent and specific locations of m^6^A marks vary between tree species and *Arabidopsis*, depending on their unique life cycles and stress conditions. For instance, salt stress in *Arabidopsis* triggers increased m^6^A deposition in the 5′- and 3′-UTRs but not in the coding regions [32]. Conversely, increased m^6^A levels in the coding region of moso bamboo promote lateral root growth [23]. This suggests that m^6^A modifications are not randomly distributed across transcripts, but specific RRACH motifs are selectively targeted for m^6^A modification, despite every transcript comprising several RRACH motifs [2].

*Differential gene architecture.* Although m^6^A is evolutionarily conserved across plant taxa, the total number of *cis-*acting and *trans*-acting elements in m^6^A-related genes differs between *Arabidopsis* and trees. Moreover, gene structures, including the numbers of exons and introns, 5′-UTR and 3′-UTR lengths, numbers of domains and motifs, number of amino acids, cellular localization, signaling, and the number of transcripts, vary between *Arabidopsis* and trees (Supplementary Data Table S3). These differences suggest that the regulation of m^6^A expression varies between these two groups of plants.

### Similarities between *Arabidopsis* and trees in m^6^A

Despite their distinct m^6^A patterns and stoichiometry, *Arabidopsis* and trees share some fundamental similarities in m^6^A mechanism.

*Conserved core m6A machinery.* The core m^6^A machinery, including WERs, is conserved between tree species and *Arabidopsis*, suggesting that the fundamental mechanism of recognizing adenosine residues and catalyzing m^6^A is likely conserved between *Arabidospis* and trees. This similarity also applies to m^6^A-mediated gene regulation, which influences gene expression, mRNA stability, translation efficiency, and splicing patterns [2, 23, 24, 45].

*Shared m6A biological functions.* Certain m^6^A biological functions, such as increased trichome density and enhanced root development, are shared between tree species (e.g. poplar and bamboo) and *Arabidopsis* [15, 23, 33].

*m6A detection technologies.* The methods are currently universal across various organisms, including trees, *Arabidopsis*, humans, and animals [48] (Table 2). There are no specialized detection techniques specifically developed for trees.

## Challenges in studying m^6^A modifications in tree species

*Arabidopsis* has been extensively studied to elucidate the mechanisms of m^6^A modification. However, m^6^A modification in tree species presents unique features and challenges compared with *Arabidopsis*. Further, while *Arabidopsis* is a valuable model plant, there is a need for a model plant specifically for forestry and horticultural research, such as poplar, to emphasize the importance of m^6^A in trees [131–133]. Below we present the challenges involved in gaining a better understanding of m^6^A modifications in tree species and suggest possible ways to overcome existing limitations.

*Comparative studies and functional roles.* Understanding the differences and similarities in the m^6^A mechanism between tree species and *Arabidopsis* can help us to identify the conserved and divergent features of the m^6^A pathway in trees as there are no comparative studies between trees and *Arabidopsis*. Characterizing tree-specific m^6^A WERs, as well as determining the functional roles of m^6^A in different tree species, are priorities.

*Functional characterization of m6A pathway genes.* Functionally characterizing m^6^A pathway genes in tree species, including their tissue-specific expression patterns and roles in plant development and stress responses, is vital for a comprehensive understanding of m^6^A-mediated gene regulation in trees.

*Manipulating m6A levels.* Developing efficient methods to manipulate m^6^A levels in tree species is essential for understanding the functional implications of m^6^A modifications and for developing m^6^A-based biotechnological tools for tree improvement. This includes exploring the potential of m^6^A-based gene editing tools to develop mutant trees with desirable traits, such as improved resistance to pests and diseases or increased tolerance to abiotic stresses.

*Molecular mechanisms and interplay.* Elucidating the molecular mechanisms governing the recognition of the RRACH motif by m^6^A writers in plants is a pivotal step in understanding m^6^A modification. Investigating the crosstalk between m^6^A and other epigenetic modifications, such as DNA methylation, offers valuable insights into the interplay of different regulatory mechanisms. In addition, exploring the role of m^6^A in the regulation of alternative splicing in trees could provide insights into how m^6^A regulates gene expression and tree development.

*Computational tools and resources.* Existing computational tools and resources are mostly used for humans and *Arabidopsis* (Supplementary Data Tables S1 andS2). Refining the tools to analyze m^6^A data in tree species can help generate complex m^6^A datasets for tree species and identify m^6^A sites, predict m^6^A targets, and provide insights into the functional implications of m^6^A modifications.

By addressing these challenges, we can underscore the importance of m^6^A in tree species, given their economic and ecological significance, and pave the way for a more comprehensive understanding of m^6^A roles in tree biology.

## Conclusions

m^6^A has emerged as a powerful gene regulator of all eukaryotes studied to date [134, 135]. It regulates mRNA stability, translation efficiency, splicing, and RNA binding proteins, particularly under different stress conditions [136–138]. However, a major limitation still exists in the need to identify m^6^A differential methylation in a genome-wide manner. Recent technologies advancements, such as chemical digestion coupled with next-generation sequencing (NGS), DRS using Oxford Nanopore Technologies [103], and single-cell deamination adjacent to RNA modification targets (DART-seq) (scDART-seq) [139], have significantly contributed to m^6^A research [140–142]. In addition, various software tools, including EpiNano and Nanom6A, have been developed to improve m^6^A quantification at single-molecule resolution. These technologies assist in deciphering m^6^A sites accurately and quantitatively, especially in cases where the modification is presented at low levels or at multiple isoform-specific sites.

Despite advancements in m^6^A detection, progress in investigating m^6^A in trees is extremely slow. Unlike cereal crops, where m^6^A is transient, m^6^A modification has long-term consequences in trees. Consequently, trees might be a new avenue for studying the long-term implications of m^6^A modification [19, 23, 24]. m^6^A WERs play important roles in executing specific modifications, influencing growth and development and enhancing stress tolerance. For instance, PtrMTA in poplar [33] and MdMTA in apple trees [24] deposit m^6^A in mRNA and promote mRNA stability and stress tolerance. In contrast, CfALKBH5 in *C. fargesii* removes m^6^A in mRNA and regulates leaf color variations [59]. Furthermore, m^6^A regulates lignin biosynthesis by maintaining mRNA stability during the rapid growth of trees. However, key differences, such as signaling pathways and protein interactions of m^6^A, exist between tree species and *Arabidopsis*. The molecular mechanisms involved in this process remain unclear, and it is uncertain whether other m^6^A members are involved in m^6^A modification and are conserved across different species. Therefore, the identification and characterization of m^6^A regulatory proteins, as well as the corresponding genes, along with comparative studies between tree species and *Arabidopsis*, may contribute to a better understanding of m^6^A modifications in trees.

## Acknowledgements

The authors wish to thank the anonymous reviewers for their valuable time and insightful comments, which greatly improved the quality of the manuscript. We apologize to those whose original work(s) could not be included in this review owing to space limitations.

Preparation of this review was supported by a grant from the National Key Research & Development Program of China (2021YFD2200503-01), a grant from the National Natural Science Foundation of China (32071848), a grant from the Natural Science Foundation of Jiangsu Province (BK20231289), the Jiangxi ‘Shuangqian’ Program (S2019DQKJ2030), the Natural Science Foundation for Distinguished Young Scholars of Nanjing Forestry University (JC2019004), the Project for Groundbreaking Achievements of Nanjing Forestry University (202211), and a project funded by the Priority Academic Program Development of Jiangsu Higher Education Institutions. The authors are also grateful for the Young Foreign Talent Program (QN2022014012L) and the support of Metasequoia Faculty Research Start-up Funding (163100028) at the Bamboo Research Institute, Nanjing Forestry University, for the first author, M.R. K.S.R. is supported by the Dean of Faculty Fellowship, the Koshland Prize, and a Sir Charles Clore Postdoctoral Fellowship from the Weizmann Institute.

## Author contributions

M.R., K.S.R., Q.W., and S.M. planned, designed and wrote the review. M.R., K.S.R., Q.W., and M.Z. outlined and edited the review. M.R., S.M., and K.S.R. drew the images. M.R. and Q.W. made the tables. M.R., K.S.R., M.Z., A.S., Z.A., S.R., and Q.W. edited and revised the review.

## Data availability

No additional data were generated or are associated with this article.

## Conflict of interest

The authors declare that the research was conducted in the absence of any commercial or financial relationships that could be construed as a potential conflict of interest.

## Supplementary data

Supplementary data is available at *Horticulture Research* online.

## Supplementary Material

Web_Material_uhad284
