# Supplementary material for: Exploring N6-methyladenosine (m6A) modification in tree species: opportunities and challenges
Source: Hortic Res. 2023 Dec 29;11(2):uhad284. doi: 10.1093/hr/uhad284 (PMC10871907; doi:10.1093/hr/uhad284)
Supplement: Web_Material_uhad284 [file web_material_uhad284.zip › Supporting Information_HR-2023-445_R2.docx]

***Horticulture Research* Supporting Information**

Article title: Exploring *N*^6^-methyladenosine (m^6^A) modification in tree species: opportunities and challenges

Authors: Muthusamy Ramakrishnan, K. Shanmugha Rajan, Sileesh Mullasseri, Zishan Ahmad, Mingbing Zhou, Anket Sharma, Subbiah Ramasamy, and Qiang Wei

Article acceptance date:

The following Supporting Information is available for this article:

**Appendix S1.** **Technologies available for the identification and localization of m^6^A**

***Antibody-based detection***

In 2012, two independent teams described methylated RNA immunoprecipitation followed by sequencing (MeRIP-seq) [1] and m^6^A-seq [2]. Combination of enriched m^6^A-modified RNA fragments and high-throughput RNA sequencing could recognise the region of m^6^A modified sites. Furthermore, to improve the accuracy of the method, the original m^6^A-seq library was also constructed for knockout of m^6^A methyltransferase (such as *MTA* and *Ime4*) as a negative control [3, 4] to ensure that any detected m^6^A modifications are specific and reliable. MeRIP-seq offers significant advantage for *in vivo* investigation of m^6^A peaks. However, it is important to note that determining the precise location of m^6^A modifications sites at single-base resolution is not feasible because m^6^A peaks are localised within an area of about 100–200 nucleotides. Nevertheless, MeRIP-seq remains a valuable method to investigate the distribution and abundance of m^6^A, providing insights into m^6^A functional roles and regulatory mechanisms.

***UV-based detection***

In 2015, photo-crosslinking-assisted m^6^A sequencing strategy (PA-m^6^A-seq) was introduced to accurately identify m^6^A sites [5]. In this method, a photoactivatable ribonucleoside, 4-thiouridine (4SU), is incorporated into cellular mRNA near m^6^A location, which undergo immunoprecipitation using m^6^A-specific antibody. UV irradiation can covalently crosslink the photoactivatable ribonucleoside and nearby antibody. Cross-linked RNA is digested with proteinase K to dissociate the crosslink. 4-thiouridine crosslinking sites is read as cytosine as opposed to a T during RT-PCR, which was caused due to the residual peptide fragments during antibody removal. Though similar to immunoprecipitation methods, the advantage is higher-resolution map of m^6^A. However, this method cannot identify m^6^A sites without 4SU incorporation. Furthermore, construction of PA-m^6^A-seq library is complicated and requires cell pre-treatment. The PA-m^6^A-seq method is best suited for detecting m^6^A changes in cultured cells but is difficult to scale up. To address the disadvantage of incorporation of photoactivatable ribonucleosides, miCLIP (m^6^A individual-nucleotide-resolution cross-linking and immunoprecipitation) is used to map m^6^A at single-nucleotide resolution by combination with antibody directly crosslinks and high-throughput sequencing [6]. This method requires enrichment of fragments RNA molecules including m^6^A modification using immunoprecipitation, followed by UV crosslinking. Anti-m^6^A antibodies cause mutational signatures at m^6^A residues after reverse transcription. This method provides low false discovery rate and high base resolution [6]. Nonetheless, the disadvantage of this method is not quantitative [7]. Furthermore, this method has unclear sensitivity and might do not necessarily detect m^6^A only [8].

***Ligation-based detection***

DNA ligases exhibited higher specificity than DNA polymerases, and the ligated nucleotides can also be easily amplified. In 2007, a method utilizing T4 DNA ligase-based method was introduced for distinguishing m^6^A-modified RNA from unmodified RNA to quantify m^6^A [9]. More recently, T3 DNA ligase has a strong ability to accurately detect and quantify m^6^A at single nucleotide resolution. This method improved m^6^A detection sensitivity by up to 54.1-fold [10]. However, it should be noted that this method may be time-consuming.

***Gene editing-based detection***

To overcome the limitations of the methods mentioned above, DART-seq (deamination adjacent to RNA modification targets) approach [11] is introduced, which can identify m^6^A sites in cells, even in 10 ng of total RNA. APOBEC1-YTH expression leads to C-to-U deamination at m^6^A-proximal residues, which can be detected by transcriptomics [11]. The m^6^A-binding YTH domain is fused to enzyme APOBEC1, a cytidine deaminase enzyme, which induced C-to-U conversion at sites adjacent to m^6^A [11]. C-to-U editing efficiency is positively correlated with m^6^A level, which suggests that DART-seq has ability in quantifying m^6^A. However, it remains unknown that whether multiple m^6^A residues on individual RNA work in a coordinated or competing manner for C-to-U editing events.

***Metabolic labelling-based detection***

The m^6^A-label-seq is a metabolic labelling approach, which enables the detection of transcriptome-wide m^6^A modification at single base resolution [12]. The method begins by feeding cells with the ability of m^6^A methyltransferase to generate a methyl group transferred to the *N^6^* position of a specific mRNA adenosine, where S-adenosyl methionine (SAM) is a cofactor of the methyltransferase. SAM is synthesised from methionine and adenosine triphosphate, which is catalysed by methionine adenosine transferase. This process converts allyl-methionine to allyl-SAM or its selenium homologue allyl-SeAM. Consequently, mRNA adenosine sites, supposed to be m^6^A-modified, could be converted to *N^6^*-allyladenosine (a^6^A) by native m^6^A methylation enzymes. Moreover, a^6^A induces the formation of cyclized *N^1^*, *N^6^*-cyclized adenosine (cyc-A) through the iodination-induced cyclization reaction, resulting in base-pair mismatch during reverse transcription. By utilizing sequencing and bioinformatics tools, the m^6^A map at single-base resolution can be generated [12]. Similarly, S-adenosyl-L-methionine (AdoMet), a direct chemo-biological m^6^A detection method, was developed using a synthetic analogue of AdoMet [13]. However, feeding cells with a methionine analog for a long time leads to cellular stress, and library construction is a time-consuming and expensive process.

**Table S1**. List of software for m^6^A identification using Nanopore direct RNA sequencing-based algorithms.

| **Software package** | **Feature level** | **Re-squiggle algorithm** | **Model/Method** | **Advantages** | **Disadvantages** | **Species** | **GitHub repository** | **References** |
| --- | --- | --- | --- | --- | --- | --- | --- | --- |
| Tombo (Nanoraw) | Signal intensity | Tombo | Kolmogorov-Smirnov test (Nonparametric test) | Detecting m^6^A sites at every read to its corresponding raw signal by comparing against samples without m^6^A modification. PCR sample comparison, *de novo* model comparison, and alternative model comparison | The requirement of m^6^A-free control samples, lower accuracy compared to other tools, the prevention of accurate single-molecule detection and unable to specify the modification type | *In vitro* transcribed (IVT) RNAs of *E. coli* | <https://github.com/nanoporetech/tombo> | [14] |
| EpiNano- support vector machines (SVM) | Signal intensity, basecalling error | Not required squiggle alignments | Machine learning | Provides a qualitative m^6^A profile by detecting modified bases through increased mismatches around the modified RNA base due to decreased basecalling qualities using SVM | Unable to distinguish only m^6^A sites due to other modification, such as m^1^A and the requirement of fully modified and unmodified IVT RNAs | Yeast and IVT RNAs | <https://github.com/enovoa/EpiNano> | [15, 16] |
| MINES | Signal intensity | Tombo | Machine learning | m^6^A assigned to more than 13,000 previously unannotated DRACH sites, offers isoform-level resolution for 2225 genes and predict m6A at single nucleotide resolution | Detecting m^6^A sites only in four types of sequence context (AGACT, GGACA, GGACC, and GGACT) by fraction modification values with the random forest method using methylation sites from miCLIP data | Human | <https://github.com/YeoLab/MINES> | [17] |
| DiffErr  (differr_nanopore_DRS) | Basecalling error | Not required squiggle alignments | Differential tests | Mapping of m^6^A in full-length mRNAs using a low-modification control and transcriptome-wide identification. Detection of transcription-start sites, alternative splicing, and poly(A) tail length and site selection. | Unable to consider the effects of modification on the raw signal, false positives, and the accuracy depends on basecalling, increased read depth, and long-read alignment | *Arabidopsis* | <https://github.com/bartongroup/differr_nanopore_DRS> | [8] |
| ELIGOS | Basecalling error | Not required squiggle alignments | Differential tests | Accurate prediction of m^6^A for the DRACH/RRACH motif at single nucleotide resolution by comparing the error profile between native RNA sequences and a reference sequence and directly identifying differential expression of m^6^A under different conditions | Unable to distinguish only m^6^A sites due to other modification and mainly identify only the location of RNA modifications. Unable to consider the effects of modification on the raw signal and the requirement of the RNA background error profile or m^6^A-free control samples | Human, IVT RNAs and Yeast | <https://gitlab.com/piroonj/eligos2> | [18] |
| DRUMMER | Basecalling error | Not required squiggle alignments | Differential tests | Detecting m^6^A sites at single nucleotide resolution by comparative profiles of basecall error rates at both exome and isoform level and long read length assignment without ambiguous | Execution fails due to the bam-readcount dependency is unstable and isoform mode is slower. The effects of modification on the raw signal are unconsidered. | Human | <https://github.com/DepledgeLab/DRUMMER> | [19] |
| Nanom6A | Signal intensity, dwell time | Tombo | Machine learning (XGBoost) | The detection of m^6^A sites within RRACH 5-mers at single-base high resolution, without the use of m^6^A-deficient mutants, and the detection of m^6^A differential expression between different samples | The training data contains only 130 sites of “RRACH” motif that is not sufficient for studying m^6^A with high accuracy and the performance of the algorithm depends on the specific dataset and experimental conditions | *Arabidopsis*, Human, IVT RNAs and poplar | <https://github.com/gaoyubang/nanom6A> | [20] |
| EpiNano-Error | Basecalling error, dwell time | Nanopolish | Differential tests | Updated version of EpiNano (stand-alone) to predict m^6^A from both base-called FASTQ (mismatches, deletions, insertions) data and raw FAST5 between two samples | Prediction of m^6^A depends on the two conditions/samples studied, unable to predict m^6^A by *de novo*, false positives and the requirement of wild type and knockout RNAs | Yeast and IVT RNAs | <https://github.com/enovoa/EpiNano> | [16] |
| Yanocomp | Signal intensity | Nanopolish | Bayesian modelling | The detection of m^6^A site within five nucleotides to make single molecule resolution in *Arabidopsis* WT against low levels of m^6^A expression (mutant), measurement of stoichiometry and correlation of m^6^A with other RNA processing events | The detection of m^6^A sites exclusively only in 3'UTRs and the requirement of a low level of m^6^A modification. It is still in beta stage, requires several software dependencies and the signal alignment to the reference sequences (before running Yanocomp), so there are likely to be errors | *Arabidopsis* | <https://github.com/bartongroup/yanocomp> | [21] |
| xPore | Signal intensity | Nanopolish | Bayesian modelling | The accurate detection of m^6^A sites at single nucleotide resolution at low stoichiometry (higher than 25%) under different conditions without unmodified control sample, the estimation of m^6^A fraction in the cell and the quantification of m^6^A differential methylation and variance of distribution | Globally fewer detection, false positives, m^6^A misclassification due to m^1^A, mostly representing the DRACH motif, unable to detect alternative splicing and other types at each position, two filtering methods and the requirement of knockout RNAs and powerful computational processors | Human | <https://github.com/GoekeLab/xpore> | [22] |
| nanoDoc | Signal intensity, dwell time | Tombo | Deep learning | Detecting m^6^A more precisely for 81 types of 5-mers against the reference sample, classifying 5-mers with multiple types of RNA modifications and the usage of Deep One-Class and a convolutional neural network algorithms for detecting RNA modifications more accurately | Unable to provide m^6^A identity due to multiple types of RNA modifications, difficult to estimate the modification frequency, and the requirement of unmodified IVT RNAs with higher efficiency. The tool is still in beta stage and needs further development | Yeast and IVT RNAs | <https://github.com/uedaLabR/nanoDoc> | [23] |
| Nanocompore | Signal intensity, dwell time | Nanopolish | Bayesian modelling | Accurate detection of m^6^A for the DRACH motif at single nucleotide resolution against the control sample with m^6^A, provides novel insights on specific RNA molecules, automated pipeline for preprocessing steps without training data across replicates and conditions and the correction of *p*-values | No quantification, false positives and misclassification of m^6^A due to other modifications, the accuracy depends on the coverage and sample conditions, unable to detect m^6^A sites at very low coverage, the requirement of knockout or knock-down or modified and unmodified IVT RNAs with higher efficiency | Human and yeast IVT RNAs | <https://github.com/tleonardi/nanocompore> | [24] |
| DENA | Signal intensity | Tombo | Deep learning | Accurate detection and quantification of m^6^A (detected by miCLIP) at single nucleotide resolution and on different isoforms for RRACH 5-mers and trained data covers over 3000 m^6^A sites with different combinations of sequence context. | The requirement of training data covering naturally occurring m^6^A patterns covering entire population and m^6^A-deficient, the requirement of adding poly(A) tails to the 3’-end for m^6^A detection on lncRNAs and high-performance computational requirements. | Human and *Arabidopsis* | <https://github.com/weir12/DENA> | [25] |
| JACUSA2 | Variant calling, Basecalling error | Nanopolish | Machine learning | Detection and quantification of m^6^A site with high accuracy across multiple samples from different types of sequencing data and capturing complex read signatures (insertions, deletions, etc.) without training data or prior knowledge | False positives, unable to detect m^6^A sites at very low concentrations or when sequencing data are of poor quality or insufficient, and the requirement of a reference  sequence and a significant amount of computational process | Human | <https://github.com/dieterich-lab/JACUSA2> | [26] |
| m^6^Anet | Signal intensity | Nanopolish | Deep learning | More accurate detection of m^6^A probability at any site for all DRACH 5-mers representing the training data, and comparison and estimation of site-specific and global differences in m^6^A sites within a sample or across multiple samples | Detection of m^6^A sites only in  DRACH 5-mer sequence context. The accuracy is influenced by nanopore sequencing chemistry, base calling and the alignment of reference sequence to the signal | Human, *Arabidopsis* and synthetic direct RNA-Seq data | <https://github.com/GoekeLab/m6anet> | [27] |
| CHEUI | Basecalling error | Nanopolish | Deep learning  (Convolutional neural networks) | Accurate concurrent detection and differential methylation of m^6^A and m^5^C at single nucleotide resolution (9-mers) at the transcriptome-wide level in any sequence context from the same sample without control sample or knockout/knock-down | Usage of two different modules (CHEUI-solo and CHEUI-diff) to predict the modifications, m^6^A misclassification due to m^1^A, false positives and the requirement of fully modified and unmodified IVT RNAs | IVT RNAs, Human | <https://github.com/comprna/CHEUI-public> | [28] |

**Table S2**. List of RNA modification databases that includes *Arabidopsis* RNA and m^6^A modifications.

| **Database** | **Descriptions** | **References** |
| --- | --- | --- |
| MODOMICS | It is a centralised resource with comprehensive information on RNA modifications, their common names, chemical structure and biosynthetic pathways, the location of RNA modification and modifying enzymes. It is linked to the RCSB protein database. <http://genesilico.pl/modomics/>. | [29] |
| RNAMDB | It serves as a focal point for 109 known RNA modifications and includes their common names, chemical structure and symbol, elemental composition and mass, phylogenetic source, etc.  <http://rna-mdb.cas.albany.edu/RNAmods/>. | [30] |
| RNAmod | It is an interactive web-based platform for automatic annotation, analysis and visualization of mRNA modifications of 21 species, including *Arabidopsis*, and RNAmod also shows the distribution of RNA modifications, locations of RNA-binding proteins (RBPs) and modified gene features. <https://bioinformatics.sc.cn/RNAmod/>. | [31] |
| RMVar  (m6Avar) | It is an updated version of m6Avar and contains 16,78,126 RNA modification-associated variants for nine types of RNA modification, including m^6^A. <http://rmvar.renlab.org/>. | [32] |
| RNAWRE | It is a dedicated database of RNA modification writers, readers and erasers, and stores 2045 manually curated enzymes for six types of RNA modifications, including m^6^A. RNAWRE also helps BLAST search in other species. <http://rnawre.bio2db.com/>. | [33] |
| RCAS | It is an R package designed in a simple manner for functional analysis of regions of interest in the context of the transcriptome. It can be used for functional enrichment analysis and motif identification.  <https://rcas.mdc-berlin.de/>. | [34] |
| REDIdb | It is a dedicated database for RNA editing and modification in plant organelles, with 26,618 RNA editing events explicitly distributed across 85 complete organelle genomes and 281 organisms.  <http://srv00.recas.ba.infn.it/redidb/index.html>. | [35] |
| RMBase | It is a comprehensive database for transcriptome-wide RNA modifications with RBPs for different types of RNA modifications from 13 species. RMBase contains ∼1 373 000 m^6^A, ∼5400 m^1^A, ∼9600 Ψ, ∼1000 m^5^C, etc., and shows relationships between RNA modification sites and microRNA target sites. <http://rna.sysu.edu.cn/rmbase/>. | [36] |
| REPIC | This is a complete database for m^6^A and epigenome with cell or tissue specific sites from 11 organisms. <https://repicmod.uchicago.edu/repic>. | [37] |
| m6A-Atlas | This is a dedicated database for m^6^A with 4,421,62 reliable m^6^A sites and offers features for the conservation of m^6^A. It also supports site-specific interaction with the m^6^A machinery.  [www.xjtlu.edu.cn/biologicalsciences/atlas](http://www.xjtlu.edu.cn/biologicalsciences/atlas). | [38] |
| M6A2Target | It is a dedicated database of m^6^A writers, readers and erasers and their gene targets with two different modules: validated targets and potential targets. It also provides a user-friendly interface.  <http://m6a2target.canceromics.org>. | [39] |
| DirectRMDB | It is the first Oxford Nanopore Technologies (ONT)-based database of quantitative RNA modification profiles. It covers 16 types of modifications with a total of 904,712 modification sites in 25 species and provides an isoform-specific overview of RNA modifications including m^6^A.  <http://www.rnamd.org/directRMDB/>. | [40] |

## References

1. Meyer KD, Saletore Y, Zumbo P et al. Comprehensive analysis of mRNA methylation reveals enrichment in 3' UTRs and near stop codons. *Cell*. 2012;**149**:1635-1646 <http://dx.doi.org/10.1016/j.cell.2012.05.003>.

2. Dominissini D, Moshitch-Moshkovitz S, Schwartz S et al. Topology of the human and mouse m^6^A RNA methylomes revealed by m6A-seq. *Nature*. 2012;**485**:201-206 <http://dx.doi.org/10.1038/nature11112>.

3. Anderson SJ, Kramer MC, Gosai SJ et al. *N^6^*-methyladenosine inhibits local ribonucleolytic cleavage to stabilize mRNAs in *Arabidopsis*. *Cell Rep.* 2018;**25**:1146-1157.e3 <http://dx.doi.org/10.1016/j.celrep.2018.10.020>.

4. Schwartz S, Agarwala SD, Mumbach MR et al. High-resolution mapping reveals a conserved, widespread, dynamic mRNA methylation program in yeast meiosis. *Cell*. 2013;**155**:1409-1421 <http://dx.doi.org/10.1016/j.cell.2013.10.047>.

5. Chen K, Lu Z, Wang X et al. High-resolution *N^6^*-methyladenosine (m^6^A) map using photo-crosslinking-assisted m^6^A sequencing. *Angew Chem Int Ed Engl*. 2015;**54**:1587-1590 <http://dx.doi.org/10.1002/anie.201410647>.

6. Linder B, Grozhik AV, Olarerin-George AO et al. Single-nucleotide-resolution mapping of m^6^A and m^6^Am throughout the transcriptome. *Nat Methods*. 2015;**12**:767-772 <http://dx.doi.org/10.1038/nmeth.3453>.

7. Wang Y-N, Yu C-Y, Jin H-Z. RNA *N*^6^-methyladenosine modifications and the immune response. *J Immunol Res.* 2020;**2020**:6327614 <http://dx.doi.org/10.1155/2020/6327614>.

8. Parker MT, Knop K, Sherwood AV et al. Nanopore direct RNA sequencing maps the complexity of *Arabidopsis* mRNA processing and m^6^A modification. *Elife.* 2020;**9**:e49658 <http://dx.doi.org/10.7554/eLife.49658>.

9. Dai Q, Fong R, Saikia M et al. Identification of recognition residues for ligation-based detection and quantitation of pseudouridine and *N^6^*-methyladenosine. *Nucleic Acids Res.* 2007;**35**:6322-6329 <http://dx.doi.org/10.1093/nar/gkm657>.

10. Liu W, Yan J, Zhang Z et al. Identification of a selective DNA ligase for accurate recognition and ultrasensitive quantification of *N^6^*-methyladenosine in RNA at one-nucleotide resolution. *Chem Sci*. 2018;**9**:3354-3359 [http://dx.doi.org/10.1039/c7sc05233b](http://dx.doi.org/10.1039/C7SC05233B).

11. Meyer KD. DART-seq: an antibody-free method for global m^6^A detection. *Nat Methods*. 2019;**16**:1275-1280 <http://dx.doi.org/10.1038/s41592-019-0570-0>.

12. Shu X, Cao J, Cheng M et al. A metabolic labeling method detects m^6^A transcriptome-wide at single base resolution. *Nat Chem Biol.* 2020;**16**:887-895 <http://dx.doi.org/10.1038/s41589-020-0526-9>.

13. Hartstock K, Nilges BS, Ovcharenko A et al. Enzymatic or in vivo installation of propargyl groups in combination with click chemistry for the enrichment and detection of methyltransferase target sites in RNA. *Angew Chem Int Ed Engl.* 2018;**57**:6342-6346 <http://dx.doi.org/10.1002/anie.201800188>.

14. Stoiber M, Quick J, Egan R et al. *De novo* identification of DNA modifications enabled by genome-guided Nanopore signal processing. *bioRxiv.* 2017;094672 <http://dx.doi.org/10.1101/094672>.

15. Liu H, Begik O, Lucas MC et al. Accurate detection of m^6^A RNA modifications in native RNA sequences. *Nat Commun.* 2019;**10**:4079 <http://dx.doi.org/10.1038/s41467-019-11713-9>.

16. Liu H, Begik O, Novoa EM. EpiNano: Detection of m^6^A RNA modifications using Oxford nanopore direct RNA sequencing. *Methods Mol Biol.* 2021;**2298**:31-52 <http://dx.doi.org/10.1007/978-1-0716-1374-0_3>.

17. Lorenz DA, Sathe S, Einstein JM, Yeo GW. Direct RNA sequencing enables m^6^A detection in endogenous transcript isoforms at base-specific resolution. *RNA*. 2020;**26**:19-28 <http://dx.doi.org/10.1261/rna.072785.119>.

18. Jenjaroenpun P, Wongsurawat T, Wadley TD et al. Decoding the epitranscriptional landscape from native RNA sequences. *Nucleic Acids Res.* 2021;**49**:e7 <http://dx.doi.org/10.1093/nar/gkaa620>.

19. Price AM, Hayer KE, McIntyre ABR et al. Direct RNA sequencing reveals m^6^A modifications on adenovirus RNA are necessary for efficient splicing. *Nat Commun.* 2020;**11**:6016 <http://dx.doi.org/10.1038/s41467-020-19787-6>.

20. Gao Y, Liu X, Wu B et al. Quantitative profiling of *N^6^*-methyladenosine at single-base resolution in stem-differentiating xylem of *Populus trichocarpa* using nanopore direct RNA sequencing. *Genome Biol.* 2021;**22**:22 <http://dx.doi.org/10.1186/s13059-020-02241-7>.

21. Parker MT, Barton GJ, Simpson GG. Yanocomp: robust prediction of m^6^A modifications in individual nanopore direct RNA reads. *bioRxiv*. 2021;2021.06.15.448494 <http://dx.doi.org/10.1101/2021.06.15.448494>.

22. Pratanwanich PN, Yao F, Chen Y et al. Identification of differential RNA modifications from nanopore direct RNA sequencing with xPore. *Nat Biotechnol.* 2021;**39**:1394-1402 <http://dx.doi.org/10.1038/s41587-021-00949-w>.

23. Ueda H. nanoDoc: RNA modification detection using Nanopore raw reads with deep one-class classification. *bioRxiv.* 2021;2020.09.13.295089 <http://dx.doi.org/10.1101/2020.09.13.295089>.

24. Leger A, Amaral PP, Pandolfini L et al. RNA modifications detection by comparative nanopore direct RNA sequencing. *Nat Commun.* 2021;**12**:7198 <http://dx.doi.org/10.1038/s41467-021-27393-3>.

25. Qin H, Ou L, Gao J et al. DENA: training an authentic neural network model using Nanopore sequencing data of *Arabidopsis* transcripts for detection and quantification of *N^6^*-methyladenosine on RNA. *Genome Biol.* 2022;**23**:25 <http://dx.doi.org/10.1186/s13059-021-02598-3>.

26. Piechotta M, Naarmann-de Vries IS, Wang Q, Altmüller J, Dieterich C. RNA modification mapping with JACUSA2. *Genome Biol.* 2022;**23**:115 <http://dx.doi.org/10.1186/s13059-022-02676-0>.

27. Hendra C, Pratanwanich PN, Wan YK et al. Detection of m^6^A from direct RNA sequencing using a multiple instance learning framework. *Nat Methods*. 2022;**19**:1590-1598 <http://dx.doi.org/10.1038/s41592-022-01666-1>.

28. Mateos PA, Sethi AJ, Ravindran A et al. Simultaneous identification of m^6^A and m^5^C reveals coordinated RNA modification at single-molecule resolution. *bioRxiv*. 2022;2022.03.14.484124 <http://dx.doi.org/10.1101/2022.03.14.484124>.

29. Boccaletto P, Stefaniak F, Ray A et al. MODOMICS: a database of RNA modification pathways. 2021 update. *Nucleic Acids Res.* 2022;**50**:D231-D235 <http://dx.doi.org/10.1093/nar/gkab1083>.

30. Cantara WA, Crain PF, Rozenski J et al. The RNA modification database, RNAMDB: 2011 update. *Nucleic Acids Res.* 2011;**39**:D195-201 <http://dx.doi.org/10.1093/nar/gkq1028>.

31. Liu Q, Gregory RI. RNAmod: an integrated system for the annotation of mRNA modifications. *Nucleic Acids Res.* 2019;**47**:W548-W555 <http://dx.doi.org/10.1093/nar/gkz479>.

32. Luo X, Li H, Liang J et al. RMVar: an updated database of functional variants involved in RNA modifications. *Nucleic Acids Res.* 2021;**49**:D1405-D1412 <http://dx.doi.org/10.1093/nar/gkaa811>.

33. Nie F, Feng P, Song X et al. RNAWRE: a resource of writers, readers and erasers of RNA modifications. *Database (Oxford).* 2020;**2020**:baaa049 <http://dx.doi.org/10.1093/database/baaa049>.

34. Uyar B, Yusuf D, Wurmus R et al. RCAS: an RNA centric annotation system for transcriptome-wide regions of interest. *Nucleic Acids Res.* 2017;**45**:e91 <http://dx.doi.org/10.1093/nar/gkx120>.

35. Lo Giudice C, Pesole G, Picardi E. REDIdb 3.0: A comprehensive collection of RNA editing events in plant organellar genomes. *Front Plant Sci.* 2018;**9**:482 <http://dx.doi.org/10.3389/fpls.2018.00482>.

36. Xuan J-J, Sun W-J, Lin P-H et al. RMBase v2.0: deciphering the map of RNA modifications from epitranscriptome sequencing data. *Nucleic Acids Res.* 2018;**46**:D327-D334 <http://dx.doi.org/10.1093/nar/gkx934>.

37. Liu S, Zhu A, He C, Chen M. REPIC: a database for exploring the *N^6^*-methyladenosine methylome. *Genome Biol.* 2020;**21**:100 <http://dx.doi.org/10.1186/s13059-020-02012-4>.

38. Tang Y, Chen K, Song B et al. m^6^A-Atlas: a comprehensive knowledgebase for unraveling the N^6^-methyladenosine (m^6^A) epitranscriptome. *Nucleic Acids Res.* 2021;**49**:D134-D143 <http://dx.doi.org/10.1093/nar/gkaa692>.

39. Deng S, Zhang H, Zhu K et al. M6A2Target: a comprehensive database for targets of m^6^A writers, erasers and readers. *Brief Bioinform.* 2021;**22**:bbaa055 <http://dx.doi.org/10.1093/bib/bbaa055>.

40. Zhang Y, Jiang J, Ma J et al. DirectRMDB: A database of post-transcriptional RNA modifications unveiled from direct RNA sequencing technology. *Nucleic Acids Res.* 2023;**51**:D106-D116 <http://dx.doi.org/10.1093/nar/gkac1061>.
